# Nephroprotective activity of virgin coconut oil on diclofenac-induced oxidative nephrotoxicity is associated with antioxidant and anti-inflammatory effects in rats

**Published:** 2020

**Authors:** Ademola C Famurewa, Gabriel G Akunna, Joseph Nwafor, Onyebuchi C Chukwu, Chima A Ekeleme-Egedigwe, Janet N Oluniran

**Affiliations:** 1 *Department of Medical Biochemistry, Faculty of Basic Medical Sciences, College of Medicine, Alex-Ekwueme Federal University, Ndufu-Alike, Ikwo, Ebonyi State, Nigeria*; 2 *Department of Anatomy, Faculty of Basic Medical Sciences, College of Medicine, Afe Babalola University, Ado-Ekiti, Ekiti State, Nigeria*; 3 *Department of Anatomy, Faculty of Basic Medical Sciences, College of Medicine, Alex-Ekwueme Federal University, Ndufu-Alike, Ikwo, Ebonyi State, Nigeria*; 4 *Department of Biochemistry, Faculty of Science, Alex-Ekwueme Federal University, Ndufu-Alike, Ikwo, Ebonyi State, Nigeria*; 5 *Ministry of Agriculture and Rural Development, Ebonyi State Agricultural Development Programme, Abakaliki, Nigeria *

**Keywords:** Diclofenac, Virgin coconut oil, Nephrotoxicity, Antioxidants, Oxidative stress

## Abstract

**Objective:**

Diclofenac is a non-steroidal anti-inflammatory drug linked with considerable organ toxicity caused via increased generation of reactive oxygen species. We evaluated whether the antioxidant effect of virgin coconut oil (VCO) could prevent diclofenac-induced oxidative nephrotoxicity in rats.

**Materials and Methods:**

Randomized rats were pre-supplemented orally with VCO (5 or 10 ml/kg body weight) from day 1 to 24, and injected with normal saline or diclofenac (100 mg/kg) from day 22 to day 24 intraperitoneally.

**Results:**

Diclofenac significantly (p<0.05) increased serum urea and creatinine levels. Renal tumor necrosis factor-α (TNF-α) and malondialdehyde (MDA) levels markedly (p<0.05) increased, whereas renal glutathione peroxidase (GPx), catalase (CAT), and superoxide dismutase (SOD) activities considerably (p<0.05) decreased compared to normal control. Histopathological alterations were caused by diclofenac. However, treatment with oral VCO for 21 days prior to diclofenac administration, attenuated histological renal damage, and restored antioxidant enzyme activities and TNF-α levels in kidney.

**Conclusion:**

These findings revealed that VCO has potential benefits to prevent diclofenac-induced nephrotoxic damage.

## Introduction

Statistics from the world health organization (WHO) show that non-steroidal anti-inflammatory drugs (NSAIDs) are the second to acetaminophen as the most commonly used drug worldwide (Soleimanpour et al., 2016 ▶). Public concern has been raised over the increasing consumption of NSAIDs such as ibuprofen, aspirin and diclofenac obtained by prescription or through the so-called “over-the-counter” means (Kristensen et al., 2018 ▶). A number of disorders due to NSAID use at high concentrations and in chronic term have been reported (Simon and Prince, 2017 ▶). In this context, many NSAIDs induce renal toxicity, but patients rarely find them avoidable. Thus, novel approaches to alleviate NSAID side effects are worth exploration.

Diclofenac (DIF) is an NSAID non-specific inhibitor of cyclooxygenase enzymes widely used to treat dysmenorrhea, pain, and inflammatory disorders (Singh et al., 2017 ▶). However, DIF has been associated with an increased risk of cardiac arrest which is comparatively higher than other drugs (Naidoo and Swan, 2009 ▶). Systematic investigations indicated that DIF triggers gastric, intestinal, hepatic and renal toxicity (Singh et al., 2016 ▶; Blackler et al., 2014; Bolat and Selcuk, 2013 ▶; Yapar et al., 2008 ▶). Cyclooxygenase-1 and 2 are essentially expressed in the kidney. The inhibition of these enzymes was suggested to cause renal ischemia leading to renal damage and oxidative stress (Bolat and Selcuk, 2013 ▶). Furthermore, earlier studies revealed a pivotal role of oxidative stress and pro-inflammatory responses in DIF-induced hepatotoxicity (Adeyemi and Olayaki, 2018 ▶; Alabi et al., 2017 ▶; Galati et al., 2002 ▶). It is therefore conceivable that antioxidant agents that could attack or break the chain of reactive oxygen species generation, would exert beneficial health effect against DIF-induced nephrotoxicity. However, published papers have shown that synthetic and artificial antioxidant agents may promote carcinogenesis by their accumulation in the body (Park and Kim, 2017 ▶). The current trend thus favors the use of natural products to combat toxicity and pathophysiological conditions. 

Accumulating evidence shows VCO antioxidant and anti-inflammatory activities (Famurewa et al., 2018 ▶; Famurewa et al., 2017 ▶). VCO is extracted from the edible part of coconut fruit (*Cocos nucifera *L.) by a natural method without chemical bleaching or refining (Jaarin et al., 2014 ▶). VCO enhances memory, inhibits oxidative stress and pathologies for health benefits (Rahim et al., 2017 ▶; Jaarin et al, 2014 ▶; Dosumu et al., 2012 ▶). There is a growing body of evidence on the antioxidant and anti-inflammatory efficacy of VCO against organ toxicities of anticancer and antiretroviral therapy (Famurewa et al., 2018 ▶; Ogedengbe et al., 2018 ▶). To our knowledge, a report on the effect of VCO on DIF nephrotoxicity, is lacking in the published literature. Therefore, the present study was designed to investigate the nephroprotective effect of VCO oral supplementation against DIF-induced oxidative stress-mediated pro-inflammation and nephrotoxicity in Wistar rats. 

## Materials and Methods

**Drug and chemicals**

Diclofenac sodium (Olfen^TM^-50) manufactured by Acino Pharmacy, Liesberg, Switzerland, was used. The assay kits for serum creatinine, urea and renal antioxidant enzymes were procured from Randox Laboratory Ltd., UK. Thiobarbituric acid (TBA) was obtained from Hi Media Laboratories, India. All other reagents were of commercial grade.

**Animals**

Twenty-four (24) female Wistar rats (100-120 g) were purchased from Animal House Section of Alex Ekweme Federal University Ndufu–Alike Ikwo, Ebonyi State, Nigeria. The rats were housed under normal condition (12 hr light/12 hr dark) at 25 ±3°C, fed with rat chow (Vital Feeds Nigeria Ltd., Jos, Nigeria) and allowed free access to distilled water. The rats were allowed two weeks to acclimatize before experimental treatment and the animals were handled in line with the standard procedures of the National Research Council, Guide for the Care and the Use of Laboratory Animals (NRC, 1985).

**Extraction of virgin coconut oil**

The virgin coconut oil was extracted according to the method of Nevin and Rajamohan (2006). ▶ Fresh coconut fruits were purchased from a commercial market in Abakaliki, Ebonyi State and used for the extraction of VCO. The white slurry obtained from the coconut milk, was sieved using cheesecloth. The filtrate was left for 2 days (48 hr). The top part was removed and mildly heated (50^o^C) to harvest the oil. The separated oil was gently scooped and filtered into an air-tight container. 

**Experimental design**

The rats were divided into 4 groups of 6 rats by random distribution, the grouping and the design were as follows:

Group 1 (Normal control): rats received 5 ml/kg body weight normal saline daily for 24 days.

Group 2 (DIF): rats received diclofenac (100 mg/kg body weight/day, i.p.) from day 22 to day 24 (Bolat and Selcuk, 2013).

Group 3 (5VCO+DIF): rats received VCO (5 ml/kg body weight/day, orally) from day 1 to day 24 + diclofenac (100 mg/kg body weight/day, i.p.) from day 22 to day 24 (Bolat and Selcuk, 2013).

Group 4 (10VCO+DIF): rats received VCO (10 ml/kg body weight/day, orally) from day 1 to day 24 + diclofenac (100 mg/kg body weight/day, i.p.) from day 22 to day 24 (Bolat and Selcuk, 2013).

All animals (fasted) were sacrificed after 3 days of DIF i.p. administration. Blood was collected via cardiac puncture by needle and syringe into plain sample bottles. The blood samples were centrifuged (3000 g for 15 min) to separate serum which was analyzed fro creatinine and urea. The renal tissue was gently removed, washed in cold saline solution, and dried using tissue paper. The homogenization of the kidney was done in PBS (0.1 M, 1:5 w/v, pH 6.4) and centrifugation was done at 4000 g for 20 min. The kidney supernatant was separated for evaluation of lipid peroxidation, antioxidant enzyme activities and TNF-α levels. Some part of the kidney was fixed in 10% buffered formalin for histopathological examinations.

**Biochemical analyses**

The serum levels of creatinine and urea were evaluated in serum samples (4°C) by assay kits from RANDOX, as guided by the instructions of the manufacturer. The renal activity of superoxide dismutase (SOD) was evaluated by the method of Arthur and Boyne (1985) ▶. The catalase (CAT) activity was assayed by the method of Sinha (1972) ▶. Glutathione peroxidase (GPx) activity was determined by Flohe and Gunzler method (1984) ▶, while lipid peroxidation marker was assayed by measuring the level of thiobarbituric acid-reactive substances (TBARS) using the method by Wallin et al. (1993) ▶. The renal level of tumor necrosis factor-α (TNF-α) was determined by an enzyme-linked immunosorbent assay kit for rats following the manufacturer’s instructions.

**Histopathological study**

The kidney fixed in 10% buffered formalin, was dehydrated in graded levels of ethanol, cleared in xylene and embedded in molten wax. Serial sections of 5 μm thickness were stained with haematoxylin and eosin (H&E) dye, hydrated in decreasing grades of ethanol and oven-dried. The slides were viewed under a microscope to examine histological features and alterations.

**Statistical analysis**

Data were analyzed by SPSS version 22 (SPSS Inc., Chicago, IL, USA) using ANOVA followed by *post-hoc* Tukey test. Data are shown as mean±standard deviation (6 rats/group). A p value less than 0.05 was considered statistically significant.

## Results

**Effect of VCO on serum kidney function markers and renal pro-inflammatory marker**

The serum markers of kidney damage were evaluated for evaluation of renal toxicity. Table 1 shows that DIF-induced significant increases (p<0.05) in serum urea and creatinine compared to the normal control group. It was observed that VCO (5 and 10 ml/kg) prominently (p<0.05) decreased serum urea and creatinine levels compared to the DIF group. The renal pro-inflammatory marker, TNF-α, significantly (p<0.05) increased in DIF only treated rats compared to the normal control. However, supplementation of DIF-treated rats with VCO significantly (p<0.05) reduced TNF-α level compared to the DIF control rats. 

**Effect of VCO supplementation on oxidative stress markers in DIF-administered rats**

**Figure 1:**
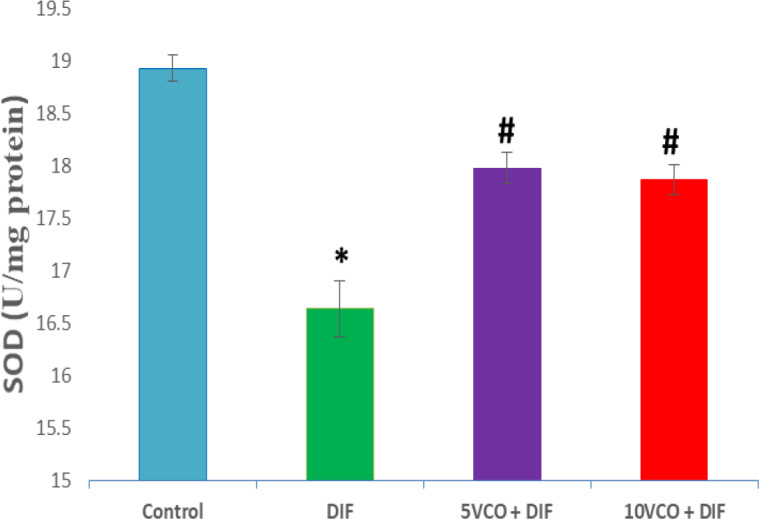
Effect of VCO supplementation on renal SOD activity in DIF-treated rats

VCO: Virgin coconut oil; DIF: Diclofenac; SOD: superoxide dismutase; Values are mean±SD (6 rats/group). *p<0.05: significant when compared with normal control group; #p<0.05: significant when compared with DIF group.

**Figure 2:**
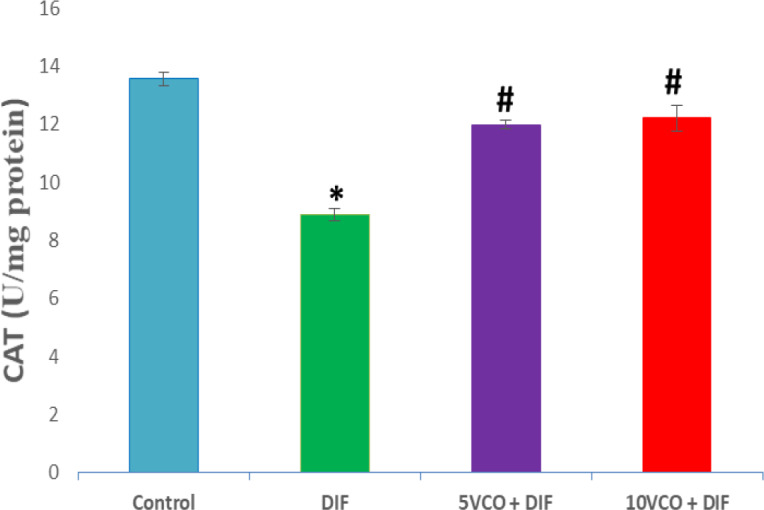
Effect of VCO supplementation on renal CAT activity in DIF-treated rats

VCO: Virgin coconut oil; DIF: Diclofenac; CAT: catalase; Values are mean±SD (6 rats/group). *p<0.05: significant when compared with the normal control group; #p<0.05: significant when compared with the DIF group.

**Figure 3:**
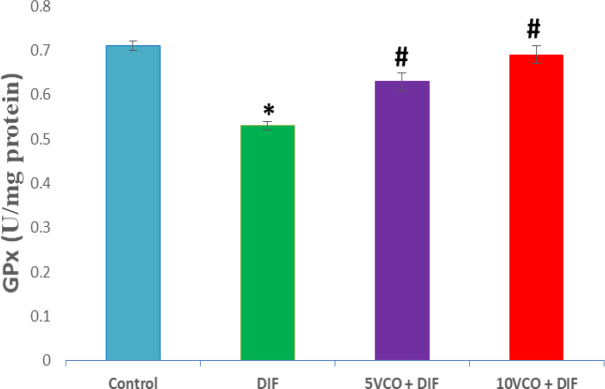
Effect of VCO supplementation on renal GPx activity in DIF-treated rats

VCO: Virgin coconut oil; DIF: Diclofenac; GPx: glutathione peroxidase; Values are mean±SD (6 rats/group). *p<0.05: significant when compared with the normal control group; #p<0.05: significant when compared with the DIF group.

**Figure 4:**
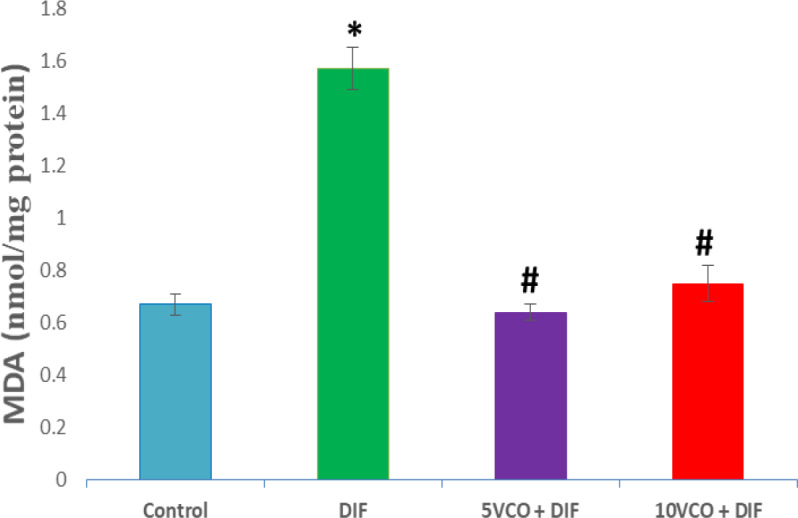
Effect of VCO supplementation on renal MDA activity in DIF-treated rats

VCO: Virgin coconut oil; DIF: Diclofenac; MDA: malondialdehyde; Values are mean±SD (6 rats/group). *p<0.05: significant when compared with normal the control group; #p<0.05: significant when compared with the DIF group.

Figures 1 to 4 depict the effects of oral VCO supplementation on SOD, CAT, GPx and MDA in DIF-treated rats. DFS injection significantly (p<0.05) reduced SOD and CAT activities in the kidney, whereas lipid peroxidation marker level, MDA, markedly increased (p<0.05) when compared to the normal control group. On the contrary, VCO supplementation (5 and 10 ml/kg) prior to and along with DIF injection, prominently (p<0.05) increased the renal SOD, CAT and GPx activities, and significantly reduced the level of MDA in comparison with the DIF group (p<0.05).

**Histopathological findings**

**Figure 5:**
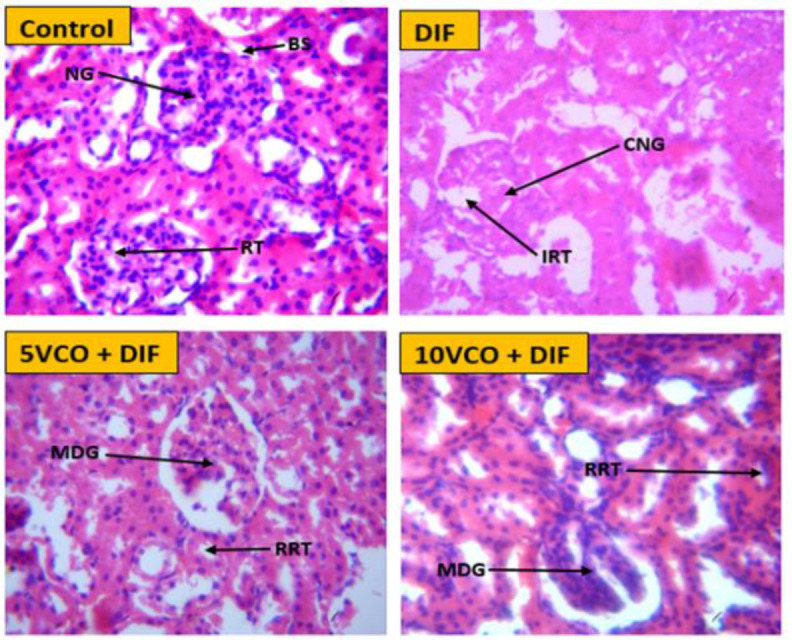
Photomicrographs of the effect of VCO and DIS on rats kidney histology. The control group showed normal kidney architecture with normal glomerulus (NG), Bowman’s space (BS) and renal tubule (RT) (Control, H-E: X400). The DIF group showed coagulative necrosis of the glomerulus (CNG), inflammatory renal tubule (IRT) (DFS, H-E: X 400). Histology of rats pretreated with VCO, showed ameliorated structure with mildly damaged glomerulus (MDG) and recovering renal tubule (RRT) (VCO 5 and 10 ml/kg, H-E: X 400). VCO: Virgin coconut oil; DIF: Diclofenac

The kidney histological photomicrographs are presented in Figure 5. The kidney of normal control rats showed normal glomerulus, Bowman’s space and renal tubule. The DIF-induced histological damage was characterized by degenerative or coagulative necrosis of the glomerulus, and inflammatory renal tubule. Histology of rats pretreated with VCO, showed ameliorated structure compared to the DIF group with mildly damaged glomerulus, and recovering renal tubule (RRT).

**Table 1:** Effect of VCO on renal function markers and tumor necrosis factor-alpha (TNF-α) levels of DIF-administered rats

**Group**	**Urea (mg/dl)**	**Creatinine (mg/dl)**	**TNF-α (pg/mg protein)**
**Normal control**	25.1±0.25	1.34±0.01	35.5±0.24
**DIF control**	32.1±1.20#	2.01±0.04#	56.0±0.58#
**5VCO+DIF**	26.9±0.25$	1.24±0. 01$	44.9±1.80$
**10VCO+DIF**	26.8±0.24**	1.19±0.04**	47.1±0.89**

VCO: Virgin coconut oil; DIF: Diclofenac; Values are mean±SD (6 rats/group).

#p<0.05: Significant when compared to the normal control group in the same column.

$p<0.05: Significant when compared to the DIF group in the same column

**p<0.05: Significant when compared to the DIF group in the same column.

## Discussion

NSAIDs typically trigger renal dysfunction. DIF has been known to be an inducer of gastric ulcers and hepato-renal toxicity in the body (Simon and Prince, 2017 ▶). The current clinical options to prevent the toxicity are not satisfactory. Use of natural products are being suggested as attractive remedies due to their insignificant side effects, low cost and availability. Supplementation of natural product to block renal DIF-induced injury could portend beneficial clinical impact on the safety of the drug. 

In the current study, DIF administration produced renal damage leading to alterations in renal cell integrity, compromised renal function, oxidative stress and pro-inflammation, confirmed by histopathological observations. The DIF-induced nephrotoxicity was evident by markedly increased serum levels of urea and creatinine. Serum urea and creatinine are sensitive and dramatic indicators of glomerular filtration rate reduction and nephrotoxicity (Shahani et al., 2016 ▶). Glomerular filtration capacity of the kidney is biochemically manifested by reduced removal of creatinine and urea from the circulating blood (Shahani et al., 2016 ▶). Hence, the levels increase in the blood. Our results here corroborate findings from previous studies that DIF exerts toxicity on renal tissue (Jerine and Sabina, 2018 ▶; Simon and Prince, 2017 ▶; Bolat and Selcuk, 2013 ▶). The nephrotoxic mechanism of DIF was shown to be associated with the DIF ability to initiate calcium influx into mitochondria leading to mitochondrial degeneration (Mingatto et al., 1996 ▶). Further, the degeneration cascades were linked with peroxidants and reactive oxygen species (Yapar et al., 2008 ▶). Mitochondrial impairment could cause renal tubular dysfunction that may trigger accumulation of urea and creatinine in the blood responsible for our findings in this study. It is interesting that VCO supplementation resisted DIF-induced nephrotoxicity. This was shown in the current study as prominently reduced serum levels of urea and creatinine in comparison to normal group rats. The histological alterations in the kidney were ameliorated by supplemented VCO. The nephroprotective effects of the two VCO doses were insignificantly varied in this study. However, studies reported nephroprotective effect of natural products on DIF toxicity (Hassan et al., 2017 ▶; Maity et al., 2012 ▶). The antioxidant phytochemical constituents of VCO may be responsible for the attenuation of DIF nephrotoxicity. The VCO constituents including ferulic, *p*-coumaric, vallinic, protocatechuic acids were suggested to underlie VCO beneficial health effects (Marina et al., 2009 ▶). Previously, these phenolic acids were reported abundantly to possess nephroprotective properties in several models of nephrotoxicity (Bami et al., 2017 ▶). 

The underlying toxicity mechanism of DIF is profoundly related to oxidative stress (Simon and Prince, 2017 ▶). Oxidative stress ensues when free radical generation overwhelms antioxidant defense system in a cell. The oxidative attack of free radicals on cells and tissue is well known to play a crucial role in toxicity development and pathophysiology of chronic diseases (Mónaco et al., 2018 ▶). Here, DIF-induced oxidative stress as demonstrated by considerable depletion in the renal activities of SOD, CAT and GPx. Consequent to the antioxidant enzyme depletion, the MDA, an important marker of lipid peroxidation, significantly increased in renal tissue. This implies that DIF-induced impairment in renal redox mechanisms. The deficit in redox balance led to oxidative damage of cell membrane lipids resulting in elevated level of MDA, which is in agreement with the observed histological alterations in kidney of rats treated with DIF alone. These findings are in tandem with earlier reports that DIF reduces activities of SOD, CAT and GPx, followed by an increase in MDA level (Jerine and Sabina, 2018 ▶; Alabi et al., 2017 ▶). This adverse effect of DIF on SOD, CAT and GPx is important for physiological processes (Traber et al, 2019). SOD, CAT and GPx are constellation of antioxidant enzymes involved in the deactivation of reactive oxygen species (ROS) generated from cells (Yazar et al., 2010 ▶). The DIF might have generated excess ROS such that it overwhelms the enzymes to produce oxidative stress. In addition, the evident oxidative stress in this study stimulates redox-sensitive cell signaling generating pro-inflammatory responses. This is indicated by increased level of TNF-α in this study. Earlier studies reported that DIF induces pro-inflammation (Jerine and Sabina, 2018 ▶; Alabi et al., 2017 ▶; Hassan et al., 2017 ▶). Oxidative stress is a prime inducer of pro-inflammation via activation of nuclear transcription factors such as nuclear factor-kappa B (NF-ĸB) (Jerine and Sabina, 2018 ▶; Bami et al., 2017 ▶). Nuclear translocation of NF-ĸB is well reported to trigger cytokine gene expression, including TNF-α found significantly increased in the current study. This oxidative stress-induced inflammation was confirmed and observed in the histology of DIF-treated rats as inflammatory renal tubules. 

Interestingly, we found that supplementation of VCO inhibited and reversed the biochemical alteration induced by DIF. VCO enhanced SOD, CAT and GPx activities in the renal tissue with consistent reduction in MDA level. Natural antioxidants and anti-inflammatory agents that inhibit ROS generation and release of inflammatory regulators like nitric oxide (NO) and cytokines, are increasingly studied (Mehta et al., 2018 ▶). To our knowledge, this is the first study to report beneficial adjuvant effect of VCO in DIF toxicity. Mounting evidence supports the inhibitory effect of VCO on oxidative stress and pro-inflammation (Famurewa et al., 2017 ▶; Vysakh et al., 2014 ▶). The VCO-induced attenuation of oxidative stress down-regulated the pro-inflammation resulting in subsequent reduction of renal TNF-α level in this study. 

Our study thus explains the DIF nephrotoxicity in female Wistar albino rats by the induction of oxidative stress and inflammation in the kidney tissue. DIF-treated rats showed alterations in renal markers, antioxidant enzymes, TNF-α and histopathology. The VCO supplementation to rats prior and along with DIF injection, reversed the altered biochemical indices and improved histopathology. Our study concludes on the nephroprotective activity of VCO against DFC-induced nephrotoxicity and pro-inflammation. Our findings may have beneficial relevance to prevent nephrotoxicity associated with DIF therapy.

## Conflicts of interest

The authors have declared that there is no conflict of interest.
